# Between a rock and a hard place: Regulation of mineralization in the periodontium

**DOI:** 10.1002/dvg.23474

**Published:** 2022-04-23

**Authors:** Natalie L. Andras, Fatma F. Mohamed, Emily Y. Chu, Brian L. Foster

**Affiliations:** ^1^ Biosciences Division, College of Dentistry The Ohio State University Columbus Ohio USA; ^2^ Division of Operative Dentistry, Department of General Dentistry, School of Dentistry University of Maryland Baltimore Maryland USA

**Keywords:** alveolar bone, cementum, extracellular matrix, mineralization, periodontal

## Abstract

The periodontium supports and attaches teeth via mineralized and nonmineralized tissues. It consists of two, unique mineralized tissues, cementum and alveolar bone. In between these tissues, lies an unmineralized, fibrous periodontal ligament (PDL), which distributes occlusal forces, nourishes and invests teeth, and harbors progenitor cells for dentoalveolar repair. Many unanswered questions remain regarding periodontal biology. This review will focus on recent research providing insights into one enduring mystery: the precise regulation of the hard‐soft tissue borders in the periodontium which define the interfaces of the cementum–PDL–alveolar bone structure. We will focus on advances in understanding the molecular mechanisms that maintain the unmineralized PDL “between a rock and a hard place” by regulating the mineralization of cementum and alveolar bone.

## 1 INTRODUCTION

**FIGURE 1:**
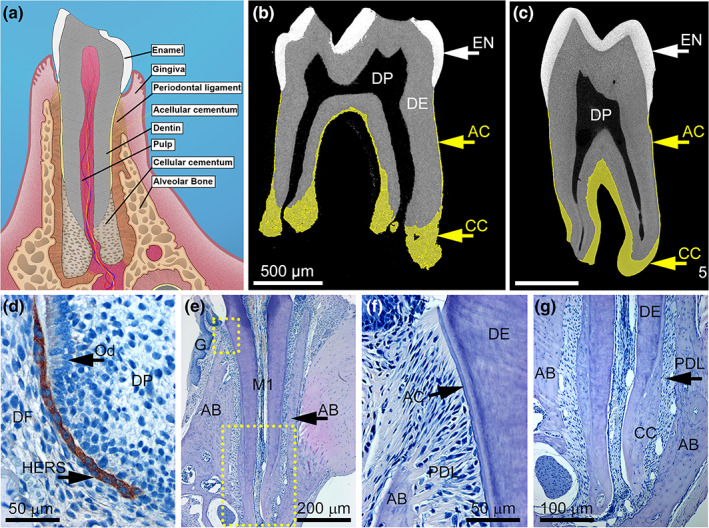
Dental and periodontal tissues. (a) Schematic of a mouse molar and supporting periodontal tissues. Distribution of cementum is shown in (b) 2D micro‐CT rendering of mouse first mandibular molar with enamel (EN; white), dentin (DE; gray), dental pulp (DP), and acellular cementum (AC) and cellular cementum (CC), both shown in yellow; and in (c) 2D micro‐CT rendering of human premolar with EN (white), DE (gray), DP, and AC and CC, both shown in yellow. (d) In a developing mouse first molar at 14 days postnatal (dpn), Hertwig's epithelial root sheath (HERS) grows apically to define the root shape (shown by pan‐keratin staining in red‐brown). Odontoblasts (Od) secrete DE and dental follicle (DF) will give rise to periodontal cells. (e) H&E stained histology section of mouse first molar (M1) in coronal view showing gingiva (g) and surrounding AB. Yellow dotted boxes indicate locations of images in panels F and G. (F) Increased magnification of M1 in panel E showing AC on the cervical root, periodontal ligament (PDL), and AB. (G) Increased magnification of M1 in panel E showing CC on the apical root, PDL, and AB. Images in a–c were reproduced with permission from   (Foster et al. 2022). Image in d reproduced with permission from Foster, Nociti, and Somerman (2022)

The periodontium is a complex structure composed of unique mineralized and nonmineralized tissues. Periodontal tissues include four main components: (a) gingiva, composed of mucosal tissue and underlying connective tissues attaching to teeth and associated bone; (b) cementum, a thin, mineralized tissue enveloping root dentin that exists primarily as acellular and cellular cementum forms; (c) periodontal ligament (PDL), an unmineralized, fibrous tissue stretching between teeth and surrounding bone; and (d) alveolar bone, a specialized type of bone responsive to dental occlusal loading (Figure 1a–c). The healthy periodontal complex provides tooth attachment, distributes forces, nourishes and invests teeth via neurovascular elements, is a source for stem and progenitor cells for dentoalveolar repair, and protects deeper periodontal tissues from microbes.

Microscopic features of cementum and bone were known from comparative anatomy studies of the 18th and 19th centuries (Foster, 2017). A number of other advances were necessary to appreciate the critical functions of periodontal tissues. G. V. Black demonstrated the complicated arrangement of periodontal attachment, including insertion of Sharpey's fibers into cementum and bone, and the presence of repair cementum. Technical advances in the 20th century, including electron microscopy and other imaging modalities, allowed new insights into cell activities during periodontal development, and studies of physiology and pathology (including periodontal disease) emphasized the functional importance of periodontal tissues. Going into the 21st century, molecular biology approaches including gene‐edited mouse models provided additional insights, although, many unanswered questions remain in periodontal biology. This review will focus on one enduring mystery: the precise regulation of hard‐soft tissue borders in the periodontium which define the interfaces of cementum–PDL–alveolar bone structure. We will focus on recent advances in understanding the molecular mechanisms regulating the mineralization of cementum and alveolar bone while maintaining PDL as an unmineralized connective tissue that lies “between a rock and a hard place.”

## 2 A BRIEF PRIMER ON PERIODONTAL DEVELOPMENT

Following tooth crown morphogenesis and prior to eruption into the oral cavity, the formation of the periodontium begins with root formation. The enamel organ gives rise to Hertwig's epithelial root sheath (HERS), which will guide root formation (Bosshardt & Schroeder, 1992; Luan, Ito, & Diekwisch, 2006). HERS cells proliferate apically to define root morphology (Figure 1d) and induce differentiation of dental papilla into odontoblasts, which produce dentin. Disassociation of HERS exposes root dentin surface areas, allowing cementoblasts access to promote cementum formation (Cho & Garant, 1988). The origin of cementoblasts remains controversial, with a classical hypothesis suggesting neural crest ectomesenchymal origin and an alternative hypothesis promoting epithelial cell transformation into cementoblasts (Bosshardt, 2005; Diekwisch, 2001; Foster, Popowics, Fong, & Somerman, 2007; Huang, Bringas Jr., Slavkin, & Chai, 2009). The slow appositional growth of acellular cementum (Figure 1e,f) throughout life occurs by progressive mineralization featuring incorporation of PDL collagen fibers as embedded Sharpey's fibers, that is, bundles of mineralized collagen fibers within the acellular cementum layer. These remain continuous with collagen fiber bundles in the forming PDL. During tooth eruption, cementogenesis transitions to produce the thicker, more rapidly forming apical root cellular cementum (Figure 1g).

Concurrent with cementum deposition and PDL fiber organization, the surrounding alveolar bone produced by osteoblasts increases in height and incorporates Sharpey's fibers into bundle bone, or alveolar bone proper, at the surface (Alfaqeeh, Gaete, & Tucker, 2013; Fleischmannova, Matalova, Sharpe, Misek, & Radlanski, 2010; Lungova et al., 2011). These activities prepare the cementum–PDL–bone periodontal attachment complex for the functional demands of occlusion (Ho et al., 2013; Sodek & McKee, 2000a).

Cementoblasts and osteoblasts produce extracellular matrix (ECM) for their respective tissues and promote the deposition of hydroxyapatite (HA), a calcium phosphate mineral deposited within and between collagen fibrils that provides mechanical strength to the tissues. ECM proteins and mineralization‐regulating enzymes will be detailed in the sections below. Due to their similar composition and mineralization, acellular and cellular cementum are sometimes described as bone‐like tissues; however, bone is highly vascular and continuously remodels in response to loading; in contrast, cementum is avascular, noninnervated, and grows by apposition, reportedly with little or no physiological turnover. The lack of physiological turnover in cementum and observations that osteoclasts more frequently target alveolar bone suggest cementoblasts and/or cementum may inhibit resorption to protect tooth root structures. While many small resorption and repair events occur on root surfaces, rapid and significant root resorption resulting from trauma, infection, rapid orthodontic tooth movement, severe periodontitis, or idiopathic mechanisms can compromise tooth structure (Chu et al., 2021; Roscoe, Meira, & Cattaneo, 2015; Wang & McCauley, 2011).

Cementum has limited ability for repair and regeneration, presumably by cementoblasts or progenitor cells residing in the PDL (Bosshardt & Sculean, 2009). The PDL and surrounding alveolar bone are responsive to mechanical loading and can remodel based on cues received from occlusal forces (Beertsen, McCulloch, & Sodek, 1997; Sodek & McKee, 2000b). Possibly, due to frequent loading from occlusal forces, alveolar bone has been described as the fastest remodeling bone in the body. Therefore, the presence of osteoclasts on alveolar bone surfaces is normal. Activities of both cementoblasts and osteoblasts must be precisely controlled to avoid ankylosis, defined as the pathological obliteration of the PDL space and fusion of cementum and alveolar bone.

This review focuses on regulators of inorganic phosphate and inorganic pyrophosphate metabolism, key ECM proteins influencing mineralization, and briefly summarizes roles of some signaling pathways in directing periodontal mineralization and maintaining the cementum–PDL–bone interface.

## 3 PHOSPHATE METABOLISM

**FIGURE 2:**
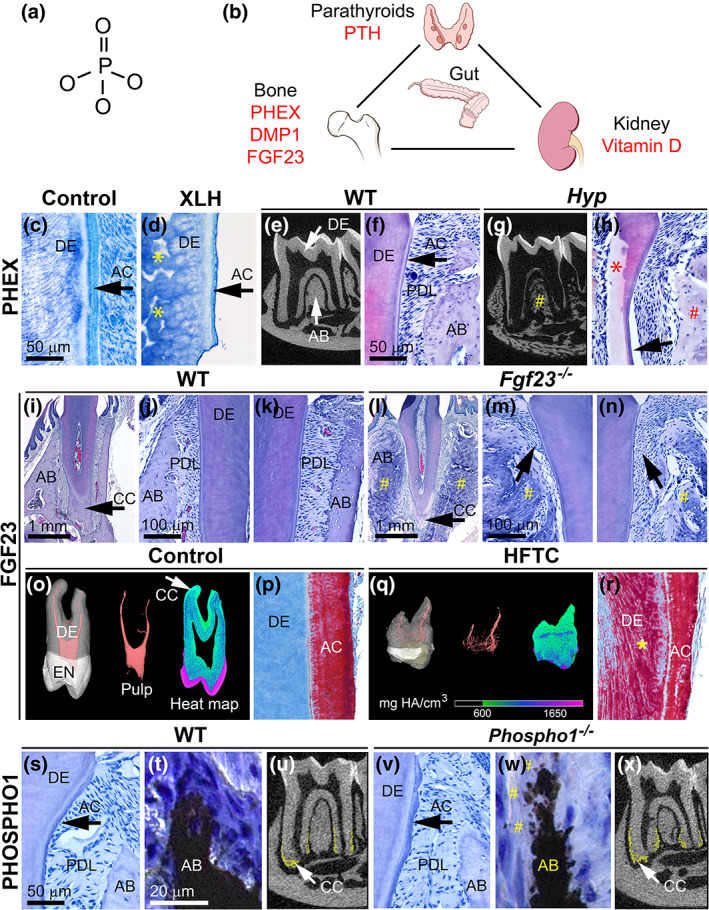
Regulators of phosphate metabolism. (a) Schematic of inorganic phosphate (P_i_), a component of hydroxyapatite (HA) mineral. (b) Schematic of major hormonal P_i_ regulators, vitamin D, PTH, and FGF23, which operate through the kidney–parathyroid–bone axis and interact with factors expressed by bone cells, including PHEX and DMP1. (c, d) Compared to healthy control primary teeth, those from individuals with XLH exhibit thin acellular cementum (AC) as well as interglobular dentin (DE) defects (*). (e–h) Compared to wild‐type (WT) mice at 42 dpn, *Hyp* mice have thin DE with interglobular defects (*), reduced AC thickness, periodontal ligament (PDL) detachment (black arrow), and regions of disorganized and hypomineralized osteoid (#) in the alveolar bone (AB). (i–n) Compared to 42 dpn WT mice, *Fgf23* ^*−/−*^ mice have dramatically expanded AB (yellow #), reduced cellular cementum (CC), and narrowing of the PDL space (black arrows). (o, q) Compared to healthy control secondary teeth, those from individuals with HFTC have altered crown and root morphologies and reduced pulp and root canals due to ectopic calcification (shown in isolated pulp images). Mineralization heat maps (color key showed in panel o) show dramatically reduced enamel (EN) and DE mineral density as well as indistinguishable DE and CC layers. (p, r) Masson's trichrome staining shows abnormal organization in root DE underling AC of teeth affected by HFTC. (s–x) Compared to WT, *Phospho1* ^*−/−*^ mice have undiminished AC and normal PDL attachment, patchy osteomalacia in AB (#) by von Kossa staining, and enlarged CC. Image in b was reproduced with permission from Foster, Chavez, Kolli, and Oldershaw (2022). Images in c and d were reproduced with permission from Clayton et al. (2021). Images in e–h were reproduced with permission from Zhang et al. (2020). Images in o–r were reproduced with permission from Lee et al. (2021). Images in s–x were reproduced with permission from Zweifler et al. (2016)

Inorganic phosphate (P_i_), abundant in the body and involved in many physiologic functions, including bone and tooth mineralization, is incorporated with calcium as HA (Figure 2a). P_i_ metabolism involves complex interactions between multiple systems beyond the scope of this review; detailed descriptions are available elsewhere (Bergwitz & Jüppner, 2010; Foster, Nociti Jr., & Somerman, 2014). Physiological levels of P_i_ (and ionic calcium; Ca^2+^) are controlled by the kidney–parathyroid–bone axis and three major hormonal regulators: (a) the active form of vitamin D (1α,25‐dihydroxyvitamin D_3,_ or 1,25D), which promotes P_i_ and Ca^2+^ absorption in the intestine; (b) parathyroid hormone (PTH), which is stimulated by hypocalcemia and adjusts Ca^2+^ levels by increasing 1,25D synthesis, renal Ca^2+^ reabsorption, and osteoclast‐mediated bone turnover to release Ca^2+^ into the bloodstream; and (c) fibroblast growth factor 23 (FGF23), which is produced by bone osteocytes and reduces circulating P_i_ by promoting renal excretion of P_i_ and reducing 1,25D levels (Figure 2b). Defects in any of these regulatory arms profoundly affect systemic P_i_ regulation. Below we highlight periodontal effects associated with (a) Phosphate‐regulating Endopeptidase Homolog, X‐linked (*PHEX*; PHEX); (b) dentin matrix protein 1 (*DMP1*/DMP1) mutations that elevate FGF23; (c) alterations to FGF23 signaling related to variants in the *FGF23 gene* or associated factors, UDP‐*N*‐acetyl‐α‐d‐galactosamine:polypeptide *N*‐acetylgalactosaminyltransferase 3 (*GALNT3*) or KLOTHO (*KL*); (d) disruptions in 1,25D metabolism; (e) dysregulation of local P_i_ regulators, Phosphatase 1, Orphan 1 (PHOSPHO1); and (f) transporters SLC20A1/P_i_T1 and SLC20A2/P_i_T2.

### 3.1 Phosphate‐regulating endopeptidase homolog, X‐linked

X‐linked hypophosphatemic rickets (XLH; OMIM# 307800), caused by inactivating mutations in Phosphate‐regulating endopeptidase homolog, X‐linked (*PHEX*), is the most common form of inherited rickets estimated at 1:20,000 births (Beck‐Nielsen, Brock‐Jacobsen, Gram, Brixen, & Jensen, 2009; Eicher, Southard, Scriver, & Glorieux, 1976). Loss‐of‐function mutations in *PHEX* lead to mineralization disturbances, including rickets and osteomalacia, due to increased FGF23, low 1,25D, renal phosphate wasting, and hypophosphatemia (Foster, Nociti Jr., & Somerman, 2014). XLH causes dental mineralization defects: thin and hypomineralized dentin with large interglobular patterns, which indicate a poorly mineralized ECM (Figure 2cd,). These defects contribute to pulpal necrosis and abscesses, which may necessitate endodontic therapy and extractions (Baroncelli et al., 2021; Chesher et al., 2018; Foster et al., 2014; Foster, Nociti Jr., & Somerman, 2014; Opsahl Vital et al., 2012).

XLH‐related hypomineralization defects extend to periodontal tissues. While relatively few reports on XLH have analyzed the periodontium, primary and secondary teeth affected by XLH exhibit thin acellular cementum (Biosse Duplan et al., 2017; Clayton et al., 2021; Figure 2c,d), and cellular cementum is hypomineralized (Coyac et al., 2017). The *Hyp* mutant mouse model of XLH carries *Phex* mutations and phenocopies aspects of XLH (Eicher et al., 1976), including enamel and dentin mineralization defects (Coyac et al., 2018; Zhang et al., 2020). *Hyp* mice exhibit thin acellular cementum, PDL detachment, and expanded, severely hypomineralized alveolar bone characterized by large swaths of osteoid and osteocyte perilacunar mineralization defects (Coyac et al., 2018; Zhang et al., 2020; Figure 2e–h). These dramatic failures of periodontal mineralization in *Hyp* mice are accompanied by abnormal mechanical properties (Zhang et al., 2020). While premature tooth loss is generally not described for XLH (extractions resulting from abscesses are most common), accumulated periodontal defects likely contribute to increased incidence of periodontal disease later in life in individuals with XLH (Biosse Duplan et al., 2017).

### 3.2 Dentin matrix protein 1

Mutations in dentin matrix protein 1 (*DMP1*), an ECM protein in the Small Integrin‐Binding LIgand N‐linked Glycoprotein (SIBLING) family (described in more detail below; Fisher & Fedarko, 2003), are associated with autosomal recessive hypophosphatemic rickets type 1 (ARHR1; OMIM# 241520). Despite their different genetic origins, ARHR1 presentation resembles XLH; pathologically elevated FGF23, reduced 1,25D, and hypophosphatemia contribute to rickets, osteomalacia, and enamel and dentin defects (Feng et al., 2006; Ni et al., 2020). *Dmp1* deleted mice (*Dmp1* ^*−/−*^) recapitulate ARHR1 (Feng et al., 2006). Similar to the *Hyp* model of XLH, *Dmp1* ^−/−^ mice exhibit thin acellular cementum, PDL detachment, osteomalacic alveolar bone, and periodontal breakdown (Foster, Nociti Jr., & Somerman, 2014; Ye, Zhang, Ke, Bonewald, & Feng, 2008). Guirado and colleagues noted disturbed expression and localization of ECM proteins (including DMP1) in dentin and pulp of *Hyp* mice, which suggests local disturbances may contribute to hypomineralization defects (Guirado et al., 2020). While in vitro addition of DMP1 protein corrected some abnormalities in XLH human dental pulp stem cell cultures, in vivo overexpression of DMP1 via the *Dspp* promoter in *Hyp* mice did not improve dentin or alveolar bone defects.

### 3.3 Fibroblast growth factor 23 and related factors

Fibroblast growth factor 23 (FGF23) is produced by osteoblasts and osteocytes in response to high circulating P_i_ and 1,25D. FGF23 binds to FGF receptor‐1 (FGFR1) and co‐receptor KLOTHO to downregulate sodium/phosphate co‐transporters NPT2a and NPT2c and 25‐hydroxyvitamin D‐1α‐hydroxylase, which converts inactive 25‐hydroxyvitamin D to 1,25D, inhibiting P_i_ reabsorption and 1,25D formation in the renal proximal tubules (Bacchetta, Bardet, & Prie, 2020; Bhattacharyya, Chong, Gafni, & Collins, 2012; Blau & Collins, 2015; Shimada et al., 2004). *Fgf23* ^*−/−*^ mice exhibit hyperphosphatemia, hypercalcemia, and elevated 1,25D, leading to severe growth retardation, skeletal deformities, and abnormal accumulation of osteoid (Bacchetta et al., 2020; Shimada et al., 2004; Sitara et al., 2004). *Fgf23* ^*−/−*^ mice exhibit ectopic matrix in pulp chambers, disorganized odontoblasts, and, in the periodontium, dramatically expanded alveolar bone with regions of hypomineralized osteoid, apoptotic osteocytes, and reduced PDL space (Figure 2i–n; Chu et al., 2010; Foster, Nociti Jr., & Somerman, 2014). While dentin and cementum appeared largely normal by histology, scanning electron microscopy revealed altered dentin and cementum of *Fgf23* ^*−/−*^ mice compared to WT, including a lack of clear cementum–dentin junction.

Several other factors modulate FGF23 signaling. Polypeptide UDP‐*N*‐acetyl‐α‐d‐galactosamine:polypeptide *N*‐acetylgalactosaminyltransferase 3 (GALNT3) glycosylates FGF23 and prevents its proteolysis, which sustains circulating levels of active FGF23 (Ho & Bergwitz, 2021). Loss‐of‐function mutations in *FGF23* or in *GALNT3* can lead to reduced intact FGF23 and increased C‐term FGF23, which causes the ectopic calcification disorder, autosomal recessive hyperphosphatemic familial tumoral calcinosis type 1 (HFTC1; OMIM# 211900; Topaz et al., 2004). Case reports for HFTC1 patients with *FGF23* and *GALNT3* mutations include dental phenotypes characterized by thistle shaped roots, obliteration of pulp chambers and root canals, enamel defects, and increased incidence of dental abscesses (Figure 2o–r; Foster, Ramnitz, et al., 2014; Lee et al., 2021). Histological and electron microscopy analysis revealed disruption of dentin tubular structure and cementum formation. In some areas, dentin and cementum could not be separated by density using microCT analysis (indicating mineralization alterations in one or both tissues), and in histological sections, both appeared disorganized with abnormally embedded cells. Similar to *FGF23* or *GALNT3* loss‐of‐function, pathological *KL* variants reduce FGF23 signaling and HFTC type 3 (OMIM# 617994), which is marked by ectopic calcification (Ichikawa et al., 2007). In a cohort of 17 HFTC patients, the patient with *KL* mutation did not exhibit the same dental phenotype as patients with *GALNT3* or *FGF23* mutations (Lee et al., 2021).

### 3.4 Vitamin D signaling

Cells of the dentoalveolar complex express the vitamin D receptor (VDR) and respond to 1,25D‐mediated signaling (Davideau, Papagerakis, Hotton, Lezot, & Berdal, 1996; Onishi et al., 2003). Vitamin D regulates P_i_ and Ca^2+^ metabolism and is affected by the derangement of FGF23 signaling (as described above for XLH, ARHR1, and HFTC), therefore we include it in this section on P_i_ metabolism. However, it should be noted that 1,25D has direct effects on cells and regulates the transcription of mineralization‐regulating genes, including (a) *BGLAP*, the gene encoding osteocalcin (OCN), (b) *IBSP*, encoding bone sialoprotein (described in more detail below; Chen, Jin, Ranly, Sodek, & Boyan, 1999; Kerner, Scott, & Pike, 1989), (c) *SPP1*, encoding osteopontin (described in more detail below; Noda et al., 1990), and likely others. Disruption of 1,25D metabolism in mice by either genetic deletion of *Cyp27b1*, which encodes the 1α(OH)ase enzyme necessary for activation of vitamin D (Davideau, Lezot, Kato, Bailleul‐Forestier, & Berdal, 2004), or ablation of the *Vdr* gene to prevent 1,25D signaling (Liu et al., 2009), resulted in reduced and disorganized alveolar bone and evidence of periodontal breakdown. More detailed summaries of vitamin D signaling and dentoalveolar tissues can be found elsewhere (Foster & Hujoel, 2018).

### 3.5 Phosphatase 1, orphan 1

Phosphate metabolism also operates locally through the actions of enzymes and transporters. Phosphatase 1, Orphan 1 (PHOSPHO1) is a member of the haloacid dehalogenase superfamily of hydrolases proposed to initiate HA deposition inside cell‐derived, membrane‐bound matrix vesicles (MV) by generating P_i_ from hydrolysis of MV membrane constituents, phosphoethanolamine and phosphocholine (Millan, 2013; Roberts, Stewart, Sadler, & Farquharson, 2004). Pathological variants in *PHOSPHO1* have not been reported in humans, but mouse model studies revealed PHOSPHO1 function in periodontal tissues. Genetic deletion of PHOSPHO1 in mice (*Phospho1* ^*−/−*^) caused dentin and alveolar bone mineralization defects, particularly when overlaid with knockout of one *Alpl* allele (McKee et al., 2013). *Alpl* and tissue‐nonspecific alkaline phosphatase (TNAP) are described in the next section. In the absence of PHOSPHO1, alveolar bone and cellular cementum show a patchy pattern of hypomineralization (Figure 2s–x; Zweifler et al., 2016), paralleling other skeletal sites (Boyde et al., 2017). Cellular cementum volume increased in *Phospho1* ^*−/−*^ versus WT mice (Mohamed et al., 2022; Zweifler et al., 2016). In contrast to other hard tissues, acellular cementum formation and periodontal attachment were not affected by loss of *Phospho1*, suggesting PHOSPHO1 functions are not critical or are overlapping/redundant in acellular cementum. Tissues affected by loss of PHOSPHO1 are those where MV have been identified during mineralization (i.e., dentin, cellular cementum, and alveolar bone), whereas MV roles have not been identified during acellular cementum formation (Takano, Sakai, Baba, & Terashima, 2000).

### 3.6 Phosphate transporters

P_i_ transport is critical for cell‐directed biomineralization activities, and encompasses P_i_ absorption from the diet, detection and response to P_i_ levels, P_i_ uptake for transportation to the mineralization front, and regulation of mineralization‐associated gene expression (Beck Jr., 2003; Beck & Beck‐Cormier, 2020; Peacock, 2021). Of P_i_ transporters detected during dentoalveolar development (Merametdjian et al., 2018), *SLC20A1* (P_i_T1) and *SLC20A2* (P_i_T2) are regarded as the most crucial. *Slc20a1* mRNA was detected in high levels in ameloblasts and minimally in other cells of developing mouse teeth (Merametdjian et al., 2018). *Slc20a2* was expressed in cells adjacent to ameloblasts and odontoblasts, and *Slc20a2* deficient mice showed enamel and dentin mineralization defects (Merametdjian et al., 2018). Due to the lack of detailed studies of the PiT1 and PiT2 in periodontal tissues, their roles in the periodontium currently remain unclear.

## 4 PYROPHOSPHATE METABOLISM

**FIGURE 3:**
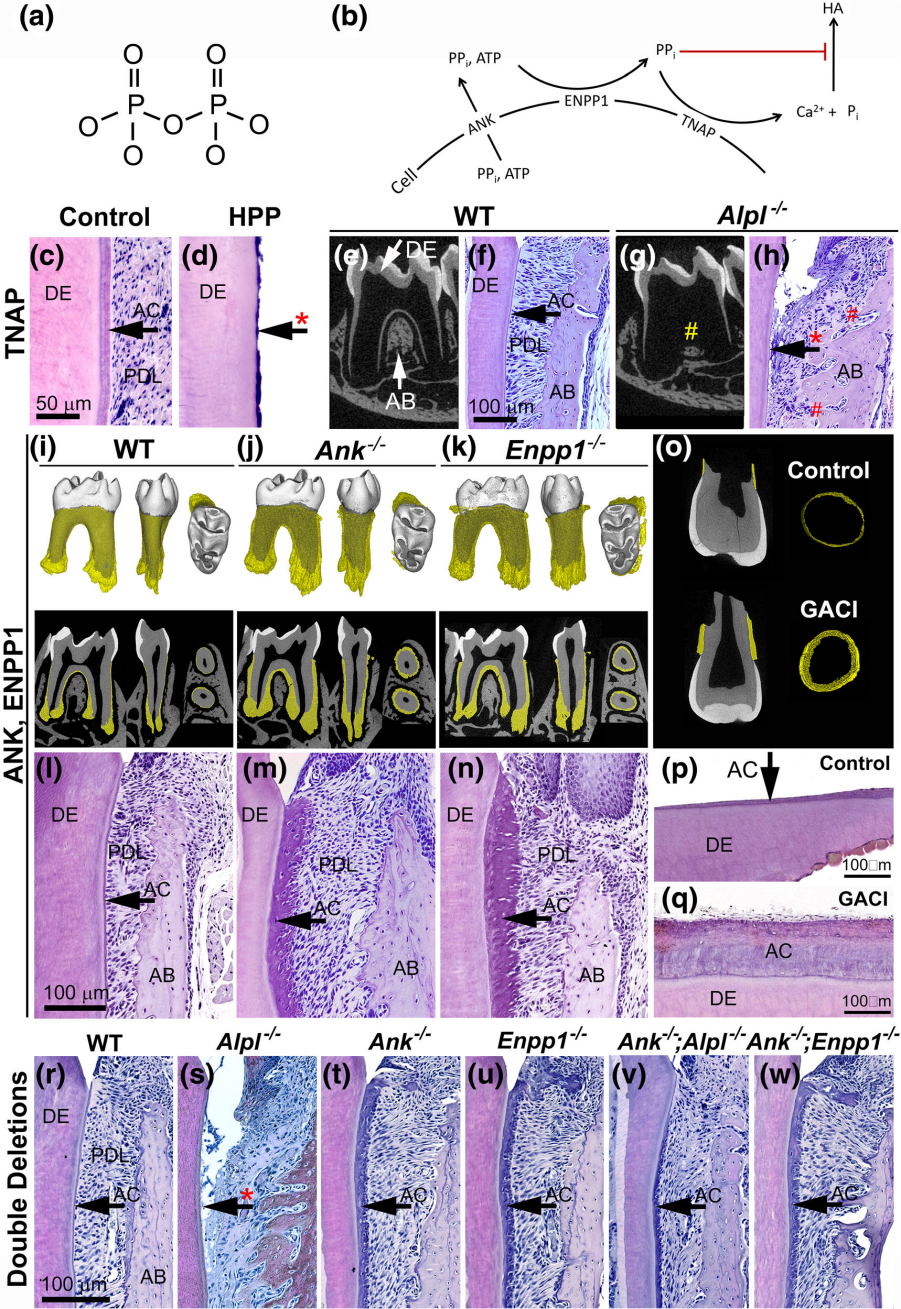
Regulators of pyrophosphate metabolism. (a) Schematic of inorganic pyrophosphate (PP_i_), a potent inhibitor of hydroxyapatite (HA) crystal growth. (b) Key elements of pericellular PP_i_ metabolism, including transport of PP_i_ and/or adenosine triphosphate (ATP) by ANK, enzymatic hydrolysis of ATP to PP_i_, and breakdown of PP_i_ to inorganic phosphate (P_i_) by TNAP that can be incorporated with calcium (Ca^2+^) to form HA. (c, d) Compared to healthy control primary teeth, those from individuals with HPP lack acellular cementum (AC), which sometimes leads to plaque accumulation (red *) deep on the root dentin (DE) surface. (e–h) Compared to wild‐type (WT) mice at 24 dpn, the *Alpl* ^*−/−*^ mouse model of severe HPP at 24 dpn has thin, hypomineralized DE (in some regions below the threshold of micro‐CT detection) and reduced alveolar bone (AB) by micro‐CT. Histology shows reduced AC (red *), periodontal ligament (PDL) detachment, and accumulated AB osteoid (#). (i–n) Compared to WT mice, molars from *Ank* ^*−/−*^ or *Enpp1* mutant mice feature dramatically increased AC. 3D (i–k, top row) and 2D (i–k, bottom row) images from micro‐CT scans show enamel in white, DE in gray, and cementum in yellow. Histology images in j–l show H&E stained sections. (o) Compared to an exfoliated primary incisor from a healthy control subject, the primary incisor from an individual with GACI from loss‐of‐function mutations in *ENPP1* exhibits increased AC (yellow), as shown in 2D reconstructions of the teeth and 3D images isolating only the AC. (p, q) H&E stained tooth sections from a control and GACI subject reveal the expanded AC. (r–w) Histology of first molar roots shows that deletion of *Ank* on the *Alpl* ^*−/−*^ background reestablishes AC. Dual deletion of *Ank* and *Enpp1* does not exacerbate hypercementosis. Images in a and b were reproduced with permission from Foster, Nociti, and Somerman (2022). Images in c and d were reproduced with permission from Chavez, Kramer, Chu, Thumbigere‐Math, and Foster (2020). Images in e and g were reproduced with permission from Kramer et al. (2021). Images in f and h were reproduced with permission from Foster, Chavez, et al. (2022). Images in i, j, and m were reproduced with permission from Ao et al. (2017). Images in k, l, n, o, p, and q were reproduced with permission from Thumbigere‐Math et al. (2018). Images in r–w were reproduced with permission from Chu et al. (2020)

Inorganic pyrophosphate (PP_i_) is an ionic counterpart of P_i_ critical for mineralization. PP_i_ is composed of two P_i_ molecules joined by a P—O—P bond that can be enzymatically hydrolyzed (Figure 3a). PP_i_ is ubiquitous in the body, present at micromolar concentrations in bodily fluids and at variable concentrations in pericellular environments (Heinonen, 2001). While P_i_ is a building block for HA crystal deposition, PP_i_ acts as a potent inhibitor of mineralization by binding and interrupting HA crystal growth (Fleisch & Bisaz, 1962; Meyer, 1984).

Critically, for biomineralization to proceed, PP_i_ can be hydrolyzed to its P_i_ constituents via pyrophosphatase activity via intracellular and extracellular enzymes (Heinonen, 2001; Millan, 2006). We focus on the three best understood PP_i_ regulators (introduced below and described in greater detail; Figure 3b), all of which play important roles in periodontal mineralization: (a) tissue‐nonspecific alkaline phosphatase (TNAP), (b) progressive ankylosis protein (ANK), and (c) ectonucleotide pyrophosphatase phosphodiesterase 1 (ENPP1).

### 4.1 Tissue‐nonspecific alkaline phosphatase

Tissue‐nonspecific alkaline phosphatase (TNAP) is encoded by the *ALPL* gene. Expressed in brain, liver, kidney, and bone, TNAP is also enriched in dentoalveolar structures, where it has roles in enamel, dentin, cementum, and alveolar bone mineralization (Zweifler et al., 2015). TNAP is best known in skeletal and dental mineralization for its enzymatic cleavage of PP_i_, which also results in the production of P_i_ ions for signaling or incorporation into HA (Hessle et al., 2002; Johnson et al., 2000; Millan, 2013; Murshed, Harmey, Millan, McKee, & Karsenty, 2005). TNAP works with at least two proteins that increase local levels of PP_i_: the progressive ankylosis protein (*ANKH*/ANK) and ectonucleotide pyrophosphatase phosphodiesterase 1 (*ENPP1*/ENPP1), discussed in more detail below. TNAP regulation of PP_i_ metabolism and periodontal mineralization has been investigated indirectly using murine cementoblast cells in culture. Exogenous PP_i_ primarily affected mineralization and indirectly influenced the expression of *Ank*, *Enpp1*, and other genes associated with P_i_, PP_i_, and mineralization (Foster et al., 2012).

Loss of TNAP function causes accumulation of extracellular PP_i_ and results in poor bone mineralization, spontaneous fractures, and premature tooth loss; this inherited condition is hypophosphatasia (HPP; OMIM# 146300, 241500, 241510; Millan & Whyte, 2016). HPP presents a wide range of symptoms of varying severity affecting nonskeletal and skeletal systems. Dentoalveolar disturbances are particularly common in HPP clinical subtypes (reviewed in Bowden & Foster, 2019; Whyte et al., 2015). Affected individuals present a spectrum of dental defects, including enamel hypoplasia, thin dentin, widened pulp chambers, defective mantle dentin mineralization, and premature tooth loss (Bowden & Foster, 2019). Tooth loss in HPP, the most frequent and sometimes sole manifestation, often occurs with minimal or no trauma in the absence of periodontal inflammation, and affected teeth are exfoliated “fully rooted”, that is, retaining significant root structure (Kramer et al., 2021; van den Bos et al., 2005). Premature tooth loss has been attributed to acellular cementum hypoplasia (Figure 3c,d; Beumer 3rd, Trowbridge, Silverman Jr., & Eisenberg, 1973; Hu et al., 2000; Kramer et al., 2021; van den Bos et al., 2005). Cementum phenotypes of HPP are variable; some prematurely exfoliated teeth from an individual with mild HPP lacked acellular cementum while others appeared to have an intact cementum layer (Kramer et al., 2021). The relationship between radicular mantle dentin mineralization defects to cementum defects (Kramer et al., 2021) and direct effects of HPP on alveolar bone mineralization and periodontal attachment are not well understood.

Studies in mice provided insights into the functions of TNAP in dentoalveolar development. Immunohistochemistry localizes TNAP expression to the developing (E15) mandibular first molar in the dental follicle, which gives rise to cementum, PDL, and alveolar bone (Bowden & Foster, 2019; Yadav et al., 2012). During root formation, TNAP localizes to newly differentiated odontoblasts producing the initial mantle dentin layer, alveolar bone osteoblasts, and presumptive cementoblasts involved in acellular cementum formation (Bowden & Foster, 2019; Foster et al., 2013; McKee, Yadav, et al., 2013; Yadav et al., 2012). Using the indoxyl‐tetrazolium salt method on undecalcified tooth cryosections (Groeneveld, Everts, & Beertsen, 1993, 1995; van den Bos & Beertsen, 1999), TNAP enzyme activity patterns were detected and proposed to be related to local variations in P_i_/PP_i_ metabolism within the periodontium.

The *Alpl* ^*−/−*^ mouse model (formerly called *Akp2* ^*−/−*^) features 75–99% reduction in circulating TNAP activity (ALP) and exhibits the spectrum of skeletal defects manifested in the severe infantile‐onset form of HPP (Fedde et al., 1999; Narisawa, Frohlander, & Millan, 1997). *Alpl* ^*−/−*^ mice exhibit mineralization defects in enamel, crown and root dentin, and alveolar bone, accompanied by lack of acellular cementum, cellular cementum mineralization abnormalities, and compromised periodontal attachment (Figure 3e–h; Beertsen, VandenBos, & Everts, 1999; Foster et al., 2012; Foster, Nagatomo, et al., 2013; Kramer et al., 2021). The absence of acellular cementum in *Alpl* ^*−/−*^ mice demonstrates the essential function of TNAP in cementogenesis. A gene‐edited knock‐in of the HPP‐associated *Alpl* A116T mutation in mice reduced ALP by only about 50% and had minimal impact on skeletal and dental mineralization; however, ALP levels correlated with acellular cementum thickness (Foster et al., 2015). Mouse models with conditional deletion of floxed *Alpl* (*Alpl* ^*fl/fl*^) carrying a *Cre* recombinase transgene under either the *Col1a1* promoter (to delete *Alpl* in osteoblasts and dental cells) or *Prx1* promoter (to delete *Alpl* in limb buds, chondrocytes, osteoblasts, and craniofacial mesenchyme) had 75% reduced ALP and severe skeletal defects (Foster et al., 2017). HPP‐associated dental defects were recapitulated, including the absence of acellular cementum, hypomineralization of alveolar bone, and periodontal breakdown.

Enzyme replacement strategies, either by daily injections or viral‐mediated gene therapy, using a mineral‐targeting form of TNAP carrying a deca‐aspartate tail (TNAP‐D_10_; asfotase alfa) have been partially successful at preventing HPP‐associated dentoalveolar defects (Foster, Nagatomo, et al., 2013; Kinoshita et al., 2021; McKee et al., 2011; Millan et al., 2008; Yadav et al., 2012). Its critical functions in periodontal development have led to studies employing TNAP for periodontal repair and regeneration. Systemic and local delivery of TNAP promoted greater periodontal repair in mice lacking BSP, which before treatment displayed severe periodontal breakdown (described in detail in the following sections; Nagasaki et al., 2021).

### 4.2 Progressive ankylosis protein and ectonucleotide pyrophosphatase phosphodiesterase 1

While TNAP reduces PP_i_ concentrations, two factors have been identified that increase local PP_i_. The progressive ankylosis gene (*Ank*; *ANKH* in humans) encodes a multipass transmembrane protein (ANK) that was reported to regulate the transport of intracellular PP_i_ to extracellular spaces (Gurley et al., 2006; Gurley, Reimer, & Kingsley, 2006; Ho, Johnson, & Kingsley, 2000), and, more recently, proposed to transport adenosine triphosphate (ATP) to the extracellular compartment (Szeri et al., 2022). Ectonucleotide pyrophosphatase phosphodiesterase 1 (*Enpp1*; ENPP1) is an ectoenzyme that produces adenosine monophosphate (AMP) and PP_i_ from adenosine triphosphate (ATP) in the extracellular milieu (Rebbe, Tong, Finley, & Hickman, 1991). Studies on mice and humans revealed acellular cementum is exceptionally sensitive to reduced PP_i_ levels resulting from ANK or ENPP1 loss‐of‐function. Loss‐of‐function mutations in *ENPP1* cause generalized arterial calcification in infancy (GACI; OMIM# 208000), whereas loss‐of‐function mutations in *ANKH* cause craniometaphyseal dysplasia (CMD; OMIM# 123000). Both disorders feature decreased systemic PP_i_ levels and marked ectopic calcification, albeit with distinct manifestations (Reichenberger et al., 2001; Ruf, Uhlenberg, Terkeltaub, Nurnberg, & Rutsch, 2005). Decreased PP_i_ from either ANK (in mice) or ENPP1 (in mice and humans) loss‐of‐function causes dramatically increased acellular cementum thickness (Figure 3i–q; Ao et al., 2017; Chu et al., 2020; Dutra, Chen, & Reichenberger, 2013; Fong, Foster, Sarikaya, & Somerman, 2009; Foster et al., 2011; Foster et al., 2012; Nociti Jr. et al., 2002; Thumbigere‐Math et al., 2018; Zweifler et al., 2015). Although the expansion of cementum appears to occur progressively throughout life, PDL space is maintained and ankylosis is prevented. Hypercementosis (overgrowth of acellular or cellular cementum) can have clinical implications; individuals with *ENPP1* mutations and GACI exhibited eruption and exfoliation abnormalities as well as reportedly slow orthodontic treatment (Thumbigere‐Math et al., 2018). Murine models, including *Enpp1* mutant mice (Wolf et al., 2018) and *Ank* mutant mice (Chen et al., 2014), showed reduced orthodontic tooth movement compared to controls.

Dual genetic ablation of PP_i_ regulators yielded additional mechanistic insights (Chu et al., 2020). Genetic deletion of the *Ank* allele in *Alpl* ^*−/−*^ mice normalized PP_i_ levels and reestablished acellular cementum formation and PDL attachment; however, cementum thickness was greater than in WT molars (Figure 3r–v). Additionally, deletion of *Ank* in *Alpl* ^*−/−*^ mice improved dentin and alveolar bone mineralization. Double deletion of *Ank* and *Enpp1* did not result in greater acellular cementum growth compared to single ablation of these genes (Chu et al., 2020). These results may have foreshadowed that ANK and ENPP1 work within the same pathway, with ANK transporting ATP for ENPP1 to hydrolyze in the extracellular space (Szeri et al., 2022). In mice lacking ANK, ENPP1, or both factors, ankylosis was avoided in part by increased osteoclast activity and remodeling of alveolar bone (Figure 3w; Chu et al., 2020). However, on a high P_i_ diet, *Enpp1* ^*−/−*^ mice developed ankylosis, which was eliminated when mice were administered an ENPP1‐Fc protein that restored PP_i_ levels and decreased hypercementosis. This result demonstrated that cementogenesis can be modulated throughout life. In all these models of altered PP_i_ metabolism, expression and localization of several cementum and alveolar bone markers were altered, which implicates these ECM factors (described in more detail below) as being important in mineralization (Chu et al., 2020). Whether these ECM changes are directly related to genetic ablation, reduced PP_i_ levels, increased cementogenesis, or a combination of these, remains to be investigated.

Developmental studies described above identified PP_i_ metabolism as a target for novel periodontal regenerative therapies. As a first proof‐of‐concept, a fenestration defect model showed that *Ank* ^*−/−*^ mice regenerated increased amounts of acellular cementum compared to WT (Rodrigues, Nagatomo, Foster, Nociti, & Somerman, 2011). Building on that study and using the same fenestration model, genetic ablation of *Ank*, *Enpp1*, or both factors increased cementum regeneration compared to controls; however, no differences were found in regenerated alveolar bone between genotypes (Nagasaki et al., 2021). Differences in cementum and alveolar bone in response to reduced PP_i_ underscore the need to consider tissue‐specific responses in strategies aiming to regenerate the entire periodontal complex.

## 5 EXTRACELLULAR MATRIX PROTEINS

ECM proteins play critical roles in regulating biomineralization in the skeleton and dentition (Boskey, 2003; George & Veis, 2008; McKee et al., 2013). While type I collagen functions as the major ECM scaffold for periodontal tissues, scores of ECM proteins and proteoglycans contribute to the precise timing, rate, and localization of HA deposition in mineralizing tissues. These include pro‐mineralization factors, which nucleate HA crystals or promote their growth, and inhibitors of mineralization, which obstruct or slow down HA crystal nucleation or growth. Below we focus on some ECM proteins that have major effects on mineralization in periodontal tissues, including collagens, bone sialoprotein (BSP), osteopontin (OPN), dentin sialoprotein (DSP), and dentin phosphoprotein (DPP).

### 5.1 Collagens

Collagens are the most abundant proteins in mammals; they define morphology, provide mechanical strength to tissues, and function as bioactive scaffolds (Ricard‐Blum, 2011). Collagen type I is a triple helix composed of collagen alpha chains encoded by *COL1A1* and *COL1A2* genes. In dentoalveolar tissues, type I collagen comprises approximately 90% of the organic ECM in dentin, cementum, and alveolar bone. Mutations in *COL1A1* and *COL1A2* are associated with osteogenesis imperfecta (OI; OMIM# 166200, 166210, 259420, and 166220 for OI types I–IV), a connective tissue disorder featuring low bone mass and increased bone fragility (Foster, Ramnitz, et al., 2014). Multiple mouse models of OI have been investigated. Mice with mutations in *Col1a1* (Brtl and *Col1a1* ^*Jrt/+*^model; Boskey et al., 2013; Eimar et al., 2016) or *Col1a2* (OIM model; Lopez Franco, Huang, Pleshko Camacho, & Blank, 2005) have dentin mineralization defects consistent with OI‐associated dentinogenesis imperfecta (DI) in humans (Foster, Ramnitz, et al., 2014). However, neither mouse models nor case reports of OI types I–IV have described substantial periodontal findings. One notable exception is a report of mild OI type IV associated with a *COL1A2* mutation where primary teeth showed ectopic calcification in dentin and cementum (Kantaputra et al., 2018).

Other types of OI are caused by deleterious mutations in genes functioning in the collagen synthesis pathway (Byers & Pyott, 2012). Bone morphogenetic protein 1 (BMP1), is an extracellular metalloproteinase involved in the posttranslational modification of collagens I–III to produce mature monomers (Kessler, Takahara, Biniaminov, Brusel, & Greenspan, 1996). Mutations in *BMP1* are associated with OI type XIII (OMIM# 614856). Simultaneous conditional ablation of *Bmp1* and mammalian tolloid‐like 1 (*mTLL1*) in mice was associated with an OI‐like condition (Muir et al., 2014). Further comparison of single and double knockout mouse models using tamoxifen‐inducible Cre^ERT2^, pointed to BMP1 as having the dominant pathologic role; BMP1 loss led to reduced cementum and alveolar bone, loss of PDL fibers, and ectopic calcification in the PDL (Wang et al., 2021).

**FIGURE 4:**
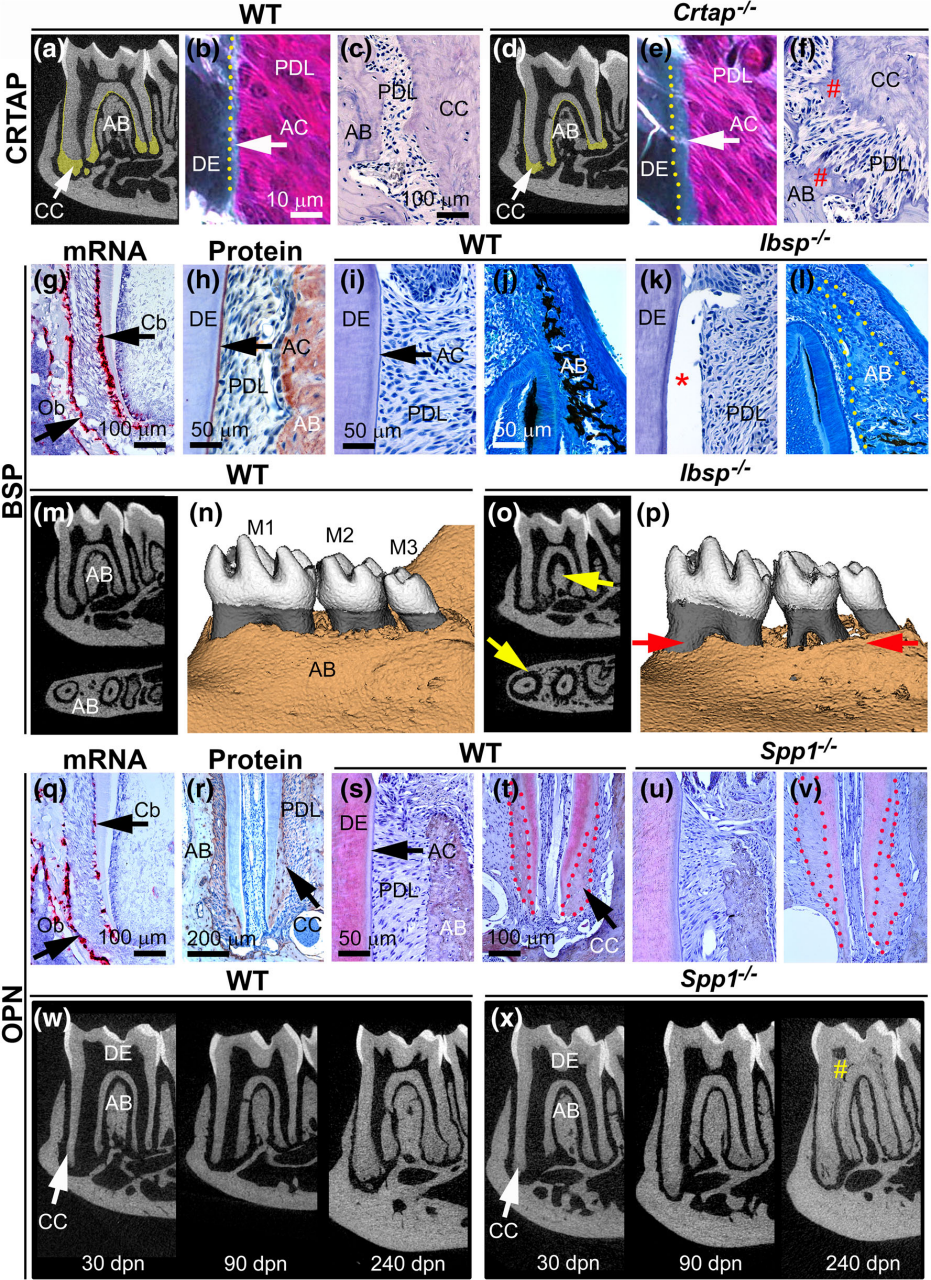
Extracellular matrix proteins involved in periodontal mineralization. (a, d) 2D sagittal sections of first mandibular molars show reduced cellular cementum (CC) and alveolar bone (AB) in *Crtap* ^*−/−*^ versus wild‐type (WT) mice at 2 months. Enamel (EN) is shown in white and dentin (DE) is shown in gray. (b, e) Goldner trichrome staining on undecalcified sections reveals thicker acellular cementum (AC; light green layer on darker green DE with dentin‐cementum junction indicated by the dotted yellow line) in *Crtap* ^*−/−*^ versus WT mice at 2 months. (c, f) H&E stain at 10–11 months shows progressive disturbance of periodontal structures in *Crtap* ^*−/−*^ mice, for example, invasion of the PDL space by inappropriate ingrowth of both AB and CC (red #). (g) During mouse periodontal development, *Ibsp* mRNA is expressed (red color) by cementoblasts (Cb) and AB osteoblasts (Ob). (h) In the completed mouse molar, BSP protein (reddish‐brown) is localized to AC and AB. (i, k) Compared to WT mouse molars at 26 dpn, *Ibsp* ^*−/−*^ mice feature reduced or absent AC and PDL detachment (red * in k). (j, l) Von Kossa staining of undecalcified histological sections reveals delayed mineralization (lack of black stain within yellow dotted region) in AB of *Ibsp* ^*−/−*^ versus WT mice at 1 dpn. (m–p) 2D and 3D micro‐CT scans of mandibles show reduced AB (yellow and red arrows) around roots of *Ibsp* ^*−/−*^ mouse molars at 60 dpn. (q) During mouse periodontal development, *Spp1* mRNA is expressed (red color) by a subset of Cb and AB Ob. (r) In the completed mouse molar, OPN protein (reddish‐brown) is localized to AC, CC, PDL, and AB. (s–v) Compared to WT mouse molars at 30 dpn, *Spp1* ^*−/−*^ mice feature relatively normal periodontal architecture, but the CC layer (red dotted outline) is enlarged. (w, x) Sagittal 2D micro‐CT renderings show increased AB and CC in *Spp1* ^*−/−*^ versus WT mice over time, as well as ectopic calcification (yellow #) in pulp of *Spp1* ^*−/−*^mouse mandibular molars with advanced age. Images in a–f were reproduced with permission from Xu et al. (2020). Images in g, q, and s–x were reproduced with permission from Foster et al. (2017). Image in h was reproduced with permission from Foster et al. (2013). Images in i, k, and m–p were reproduced with permission from Ao et al. (2017). Images in j and l were reproduced with permission from Foster et al. (2015)

Cartilage‐associated protein (CRTAP) is an endoplasmic reticulum protein working in concert with prolyl‐3‐hydroxylase‐1 (P3H1) and cyclophilin B (CYPB) to posttranslationally modify procollagen chains to facilitate folding of the triple helix (Morello & Rauch, 2010). Pathogenic mutations in the *CRTAP* gene cause OI type VII (OMIM# 610682; Barnes et al., 2006; Morello et al., 2006). *Crtap* ^*−/−*^ mice exhibit increased acellular cementum thickness, with an irregular surface topology at older ages, decreased cellular cementum formation, and substantially decreased alveolar bone volume and mineral density (Figure 4a–f; Xu et al., 2020). Ectopic calcification within the PDL and bone‐tooth ankylosis were detected in some locations, indicating dysregulated periodontal mineralization. Acellular cementum changes are consistent with electron microscopy findings in a human patient with type IV OI (Kantaputra et al., 2018); this suggests multiple forms of OI may have similar dysregulation of periodontal mineralization.

### 5.2 Bone sialoprotein

Bone sialoprotein (BSP) is a multifunctional ECM protein found in mineralized tissues of the skeleton and dentition, including bone, cartilage, dentin, and acellular and cellular types of cementum. BSP is a member of the SIBLING protein family, like DMP1 introduced above (Fisher & Fedarko, 2003), and has roles in cell attachment, cell signaling, collagen binding, and hydroxyapatite nucleation (Ganss, Kim, & Sodek, 1999). Based on multiple in vitro studies implicating it in HA nucleation and/or growth, it was hypothesized BSP had a role in regulating mineralized tissue development (Goldberg, Warner, Stillman, & Hunter, 1996; Hunter & Goldberg, 1994; Tye et al., 2003). Deletion of the *Ibsp* gene in mice (*Ibsp* ^*−/−*^ or *Bsp* ^*−/−*^) resulted in delayed mineralization initiation and smaller long bones compared to controls (Holm, Aubin, Hunter, Beier, & Goldberg, 2015; Malaval et al., 2008).

Expression of *Ibsp* by cementoblasts and the presence of BSP in acellular and cellular cementum suggested a role in cementogenesis and led to its use as a cementum marker (Figure 4g,h; Foster, 2012; MacNeil et al., 1995; M. D. McKee, Zalzal, & Nanci, 1996). *Ibsp* ^*−/−*^ mice exhibited thin acellular cementum on molar teeth, poorly embedded Sharpey's fibers, PDL detachment and disorganization, and down the growth of the junctional epithelium (Figure 4i,k; Foster et al., 2015; Foster, Soenjaya, et al., 2013; Soenjaya et al., 2015). *Ibsp* ^*−/−*^mouse alveolar bone shows large swaths of unmineralized osteoid, indicating a more severe delay in mineralization compared to long bones (Figure 4j,l), possibly due to its ectomesenchymal origin, intramembranous mode of ossification, and/or high remodeling rate (Foster, Ao, et al., 2015). Cellular cementoid accumulation was observed in *Ibsp* ^*−/−*^ mice, which indicated delayed mineralization similar to alveolar bone. These bone and cementum developmental defects contributed to rapid, progressive periodontal destruction via osteoclast resorption of alveolar bone and tooth roots (Figure 4m–p). As alveolar bone resorbed over time, molars in *Ibsp* ^*−/−*^ mice showed increasing external cervical root resorption. Acellular cementum defects also contributed to frequent incisor malocclusion and delayed incisor eruption in *Ibsp* ^*−/−*^ mice, due to the requirement of an intact periodontium in continuously erupting teeth (Soenjaya et al., 2015). Soft diet administration reduced incisor malocclusion in *Ibsp* ^*−/−*^ mice, but could not rescue the periodontal phenotype. Collectively, these studies suggest BSP is critical for cementum and alveolar bone mineralization as well as periodontal attachment and function.

Based on the studies described above, PP_i_ controllers and BSP have emerged as the most potent molecular regulators of periodontal mineralization. Potential interactions between BSP and PP_i_ metabolism were tested by genetically crossing *Ibsp* ^*−/−*^ and *Ank* ^*−/−*^ mice to determine whether decreasing PP_i_ levels would correct cementum formation and periodontal function in the absence of BSP (Ao et al., 2017). Genetic ablation of *Ank* on the *Ibsp* ^*−/−*^ mouse background successfully reestablished acellular cementum formation and periodontal function, resulting in more than a three‐fold increase in acellular cementum volume compared to WT mice. However, acellular cementum in the double knockout mice was still significantly reduced compared to *Ank* ^*−/−*^ mice, suggesting PP_i_ is more potent than BSP as a cementum regulator.

Similar findings resulted from studies in which TNAP was delivered to *Ibsp* ^−/−^ mice to a fenestration defect (Nagasaki, Nagasaki, Kear, et al., 2021). Systemic (lentivirus construct) and local delivery (addition of a recombinant protein) of TNAP substantially increased alveolar bone volume and cementum thickness in *Ibsp* ^−/−^ mice, with greater effects on PDL attachment and improved periodontal structure from local versus systemic application, despite the absence of BSP. While previous studies focused on genetic proof‐of‐concept models, this experiment showed that pharmacologic modulation of PP_i_ metabolism can overcome periodontal breakdown and accomplish regeneration.

### 5.3 Osteopontin

Osteopontin (OPN) is a member of the SIBLING protein family and is closely related to BSP (Fisher & Fedarko, 2003). OPN negatively regulates mineralization in vitro and in vivo (Boskey, Spevak, Paschalis, Doty, & McKee, 2002; Holm et al., 2014; Hunter, Hauschka, Poole, Rosenberg, & Goldberg, 1996), and is present in cementum and alveolar bone (Figure 4q,r; Bosshardt, Zalzal, McKee, & Nanci, 1998; Bronckers, Farach‐Carson, Van Waveren, & Butler, 1994; MacNeil et al., 1995; M. McKee et al., 1996; Somerman et al., 1990). Discerning OPN functions in mineralization has been complicated by its regulation of/by other factors, for example, deletion of *Alpl* increases OPN and deletion of *Ank* decreases OPN in mice (Harmey et al., 2004), while genetic ablation of *Spp1*/OPN in mice results in elevated PP_i_ levels via increased ANK and ENPP1 and reduced TNAP (Harmey et al., 2006).

OPN is hypothesized to serve as a negative regulator of periodontal mineralization (a sort of antithesis to positive regulator, BSP). A proteomic study to identify factors preventing pathological PDL calcification in *Ank* ^*−/−*^ mice identified OPN, supporting this contention (Foster et al., 2018). Analysis of dentoalveolar tissues in mice lacking OPN (*Spp1* ^*−/−*^), revealed increased volumes and/or mineral densities of dentin, cellular cementum, and alveolar bone in *Spp1* ^*−/−*^ versus WT mice (Figure 4s–x; Foster et al., 2018). However, acellular cementum growth was unaltered in *Spp1* ^*−/−*^ mice and PDL remained patent and functional, which indicated OPN is not a critical, nonredundant factor in PDL maintenance in the context of cementum and bone mineralization. This finding was reinforced by the deletion of *Spp1* in *Ank* ^*−/−*^ mice, which did not exacerbate the hypercementosis resulting from low PP_i_ (Foster et al., 2018). With greater age, some 240 dpn *Spp1* ^*−/−*^ mice showed ectopic calcification of pulp chambers (Figure 4w,x). Abnormal OPN localization is observed in dentin, cellular cementum, and alveolar bone in disorders including XLH (described above; Boukpessi et al., 2017; Coyac et al., 2017; Salmon et al., 2014). These collected observations point to pathological and physiological roles for OPN.

### 5.4 Dentin sialophosphoprotein and its products

The dentin sialophosphoprotein (*DSPP*) gene encodes two proteins arising from posttranslational enzymatic cleavage, dentin sialoprotein (DSP) and dentin phosphoprotein (DPP). Both are members of the SIBLING protein family, which includes BSP, OPN, and DMP1 (Fisher & Fedarko, 2003; Prasad, Butler, & Qin, 2010). Originally discovered in dentin, *Dspp* and its cleavage products were later reported in periodontal tissues, including cementum and alveolar bone, as well as at lower levels in long bones (Baba et al., 2004; Qin et al., 2002). Accumulated in vitro and in vivo data support DSP and DPP as positive regulators of mineralization (Prasad et al., 2010; Suzuki et al., 2009). Both genetic ablation of *Dspp* and prevention of enzymatic processing (by a knock‐in mutation preventing cleavage to functional proteins) in mice led to reduced cellular cementum, PDL detachment, alveolar bone resorption, and severe periodontal breakdown (Gibson, Jani, et al., 2013; Gibson, Zhu, et al., 2013).

## 6 SIGNALING PATHWAYS THAT REGULATE PERIODONTAL MINERALIZATION

Numerous signaling pathways influence periodontal tissue formation and biomineralization, albeit indirectly by regulating the effectors discussed above. While it is beyond the scope of this review to discuss these in great detail, we summarize findings associated with some major signaling families, including transforming growth factor β (TGFβ), bone morphogenetic proteins (BMPs), and Wnt‐β‐Catenin signaling. Additional reviews and chapters can be consulted for more detailed information on these pathways (B.L. Foster, Nagatomo, et al., 2013; Li, Parada, & Chai, 2017; and those cited below).

### 6.1 Transforming growth factor Beta

**FIGURE 5:**
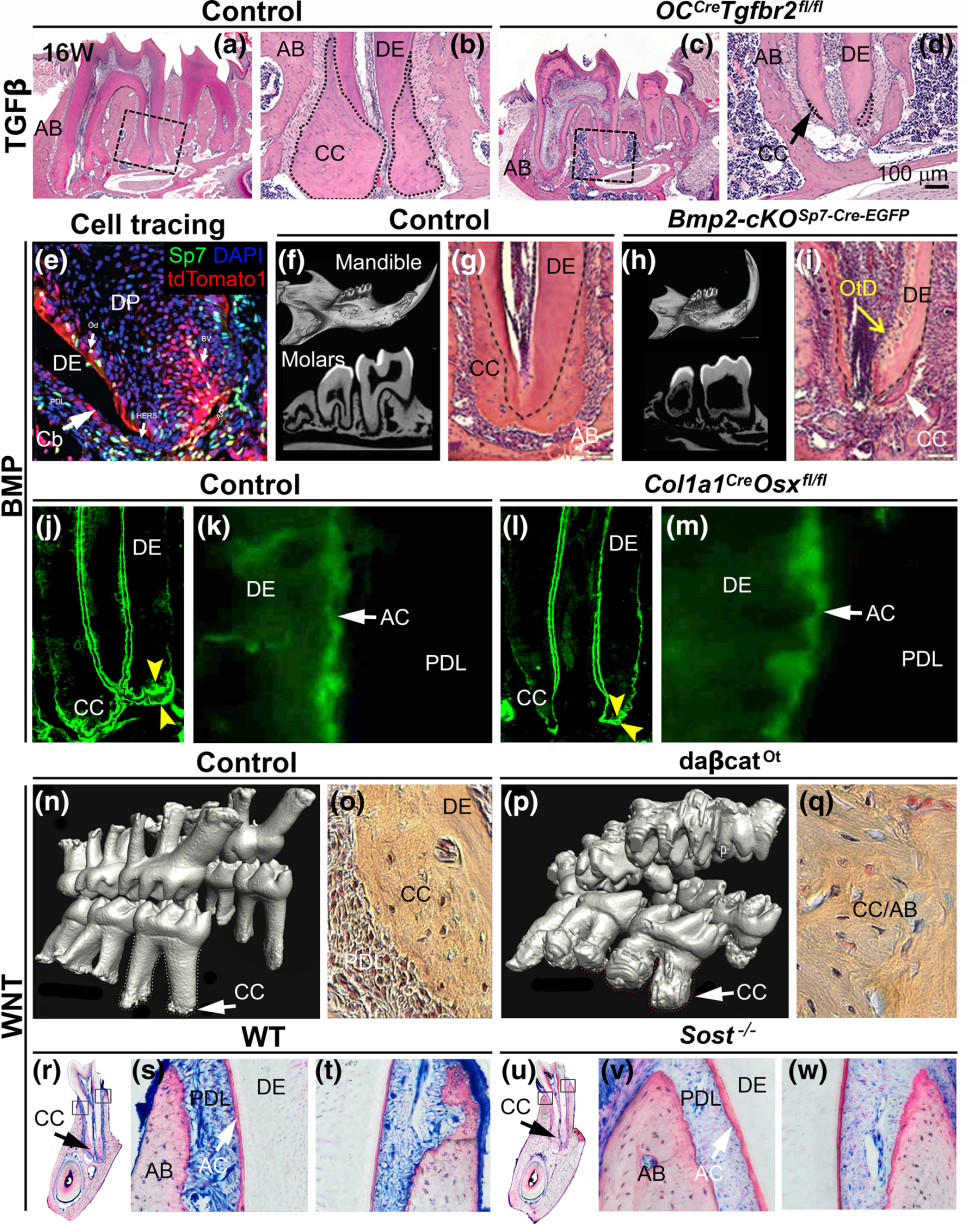
Signaling pathways directing periodontal mineralization. (a–d) Compared to *Tgfbr2* ^*fl/fl*^ controls at 16 weeks (16W), *OC* ^*Cre*^ *Tgfbr2* ^*fl/fl*^ conditional knockout mice show a marked reduction in cellular cementum (CC) in addition to disruptions in dentin (DE) and alveolar bone (AB). Boxes in a and c indicate locations of b and d. (e) Confocal microscopy showing combined lineage tracing with *Sp7*‐expressing cells (green), tdTomato1 (red) stained cells indicating *Sp7*‐mediated Cre events and cells derived from them, and DAPI‐stained nuclei (blue). (f, h) 3D and 2D micro‐CT renderings showing defective root DE, CC, and AB as well as periodontal breakdown in *Bmp2‐cKO* ^*Sp7‐Cre‐EGFP*^ conditional knockout versus littermate control (*Het* ^*Sp7‐Cre‐EGFP*;*Bmp2fx/+*^) mice at 3 months. (g, i) Histology shows failure of CC formation and disorganized osteodentin (OtD) formation in mice where *Bmp2* was conditionally deleted. (j–m) Fluorochrome‐labeled sections of molars reveal reduced mineralization rate (indicated by the distance between fluorochrome labels; yellow arrowheads) in CC in *Col1a1* ^*Cre*^ *Osx* ^*fl/fl*^ conditional knockout versus age‐matched control mice at 6 weeks. AC mineralization is less affected or unaffected by conditional deletion of *Osx*. (n, p) 3D μCT micro‐CT renderings show dramatic overgrowth of CC in daβcat^Ot^ over‐expressing versus control (Catnb^lox[ex3]/+^) mice at 42 dpn. (o, q) Pentachrome staining shows that compared to the normal CC‐PDL‐AB periodontal structure in controls, daβcat^Ot^ mice exhibit ankylosis and continuous CC/AB. (r–w) Levai–Laczko staining shows increased AC, CC, and AB in *Sost* ^*−/−*^ versus WT mice at 4 months. Images in a–d were reproduced with permission from Choi et al. (2016). Images in e–i were reproduced with permission from Rakian et al. (2013). Images in j–m were reproduced with permission from Cao et al. (2012). Images in n–q were reproduced with permission from Wu et al. (2019). Images in r–w were reproduced with permission from Kuchler et al. (2014)

The Transforming Growth Factor Beta (TGFβ) family includes over 30 cytokines that have profound effects on cell proliferation and differentiation, as well as tissue morphogenesis, homeostasis, and regeneration, via receptor‐mediated activation of SMAD transcription factors (Massague, 2012). TGFβ signaling cross‐talks with bone morphogenetic protein signaling (discussed below). TGFβ‐1 cytokine was detected in cementum, PDL, and alveolar bone of rats (Gao, Symons, & Bartold, 1998). Conditional deletion of TGFβ receptor 2 (*Tgfbr2*) in Osterix‐expressing cells resulted in defective root dentin mineralization, delayed HERS elongation and fenestration, eruption defects (Wang, Cox, Coricor, MacDougall, & Serra, 2013), and resulted in defective root morphology (Snider et al., 2021). Another approach where *Tgfbr2* was conditionally deleted under the osteocalcin promoter (*OC* ^*Cre*^ *Tgfbr2* ^*fl/fl*^) affected cementoblasts and cementocytes, which reduced cellular cementum volume and mineralization rate (Choi et al., 2016; Figure 5a–d). These changes were associated with reduced expression of transcription factor, Osterix (SP7/OSX; discussed more below), BSP, and DMP1 (discussed in previous sections).

### 6.2 Bone morphogenetic proteins

The bone morphogenetic protein (BMP) family includes at least 20 growth factors involved in embryonic patterning, many with well‐established osteogenic and chondrogenic effects via induction of progenitor cell differentiation (Graf, Malik, Hayano, & Mishina, 2016; Rosen, 2006). A subset of BMPs, including BMP‐2, BMP‐4, BMP‐7, and BMP‐9, have osteoinductive effects by inducing de novo bone formation (Kaigler, Cirelli, & Giannobile, 2006). Disruption of BMP signaling in mice via deletion of key BMP genes or overexpression of BMP inhibitors causes defects in osteoblast differentiation, as well as reduced bone volume and mineral density (Gazzerro et al., 2005; Mikic, van der Meulen, Kingsley, & Carter, 1995; Wu et al., 2003). Over‐expression of Gremlin (*Grem1*), an antagonist of BMP‐2, BMP‐4, and BMP‐7, under the *OC* ^*Cre*^ promoter, caused disruption of periodontal structures and marked enamel and dentin‐pulp defects (Nagatomo et al., 2008). Deletion of *Bmp2* in mice under control of the *Sp7* ^*Cre*^‐EGFP promoter reduced cellular cementum and alveolar bone formation as well as led to severe enamel and dentin defects (Rakian et al., 2013; Figure 5e–i). Interestingly, in both *Grem1* over‐expressing and *Bmp2* conditionally deleted mice, acellular cementum was unaffected, which suggests alternative and distinct BMP signaling patterns compared to other periodontal mineralized tissues.

Transcription factors, runt‐related transcription factor 2 (Runx2), and Osterix are essential for osteoblast differentiation, and their expression is mediated by BMP signaling (Nishimura, Hata, Matsubara, Wakabayashi, & Yoneda, 2012). Both Runx2 and Osterix are associated with alveolar bone and cementum formation (Cao et al., 2012; da Silva Sasso et al., 2021; Hirata, Sugahara, & Nakamura, 2009). Early studies of Runx2 in mice were hampered by lethality of *Runx2* gene knockout; analysis of *Runx2* heterozygous mice suggested a role for the transcription factor in tooth eruption via osteoclastogenesis, which mimicked eruption delays seen in cleidocranial dysplasia (CCD; OMIM# 119600) resulting from *RUNX2* mutations (Kreiborg & Jensen, 2018). Conditional deletion of *Runx2* in a population of Gli1^+^ odontoblast progenitors has implicated it in root formation via regulating Wnt signaling (Wen et al., 2020); however, detailed analyses of gene deletion in periodontal connective tissues have not yet been reported.

Expression of osterix closely correlated to cellular cementum formation; over‐expression of osterix under the 3.6‐kb *Col1a1* promoter (targeting genes in osteoblasts, fibroblasts, and cementoblasts) increased cellular cementum. In contrast, conditionally ablating osterix using the 2.3‐kb *Col1a1* promoter (early activation in mesenchymal tissues) or CAG‐CreER (allowing temporal control via tamoxifen injection) both reduced cellular cementum and its mineralization rate (Cao et al., 2012; Figure 5j–m). Interestingly, acellular cementum was minimally affected or unaffected by genetic editing of osterix, which highlights another fundamental difference between cells producing acellular and cellular cementum.

### 6.3 Wnt/β‐catenin signaling

The Wnt signaling pathway is an ancient and conserved signaling mechanism that regulates cell fate, polarity, and migration, as well as directs patterning and organogenesis during embryonic development; Wnt signaling also has important roles in bones and teeth (Duan & Bonewald, 2016; Huybrechts, Mortier, Boudin, & Van Hul, 2020; Li et al., 2017). The Wnt family includes 19 glycoproteins that have multiple mechanisms for signaling. The “canonical” Wnt signaling pathway occurs through binding of secreted Wnt proteins to cell surface receptors in the frizzled (FZD) family and low‐density lipoprotein receptor‐related protein (LRP) 5/6 co‐receptors. Binding results in stabilization and nuclear translocation of transcription factor, β‐catenin. Wnt signaling is active in periodontal tissues, notably in regions of proliferation and potential progenitor cells in the PDL (Rooker, Liu, & Helms, 2010), but has also been reported in mineralizing cells, including ameloblasts, odontoblasts, cementoblasts, and osteoblasts using reporter mice in lineage tracing studies (Suomalainen & Thesleff, 2010; Yin et al., 2015).

Functional importance of Wnt signaling in periodontal biology was tested in several gene edited mouse models, some of which increase Wnt signaling while others decrease Wnt signaling. Constitutive activation of the LRP5 co‐receptor in Lrp5(ACT) mice resulted in increased osteogenic expression and alveolar bone formation and concomitant reduction in osteoclastogenesis, which reduced PDL width (Lim, Liu, Mah, Yin, & Helms, 2015). Stabilized β‐catenin in mice (Daβcat^Ot^) under control of the *Dmp1* promoter increased Wnt signaling in osteocytes and cementocytes and caused dramatically increased alveolar bone and cellular cementum production (Figure 5n–q; Wu et al., 2019). Intriguingly, increased Wnt in this model resulted in loss of PDL width and regions of bone‐tooth ankylosis. Using the OC promoter to stabilize β‐catenin in mice (*Catnb* ^*lox(ex3)/+*^) also led to excessive cementum as well as increased and dystrophic dentin in association with increased cementoblast expression of *Ibsp* and *Col1a1* (Kim et al., 2011). Wnt1 is a Wnt ligand that is an important regulator of cranial neural crest cells (progenitors for the ectomesenchymal cells of the craniofacial region), and mutations are associated with OI type XV (OMIM# 615220). Inducible Wnt1 expression via the *Col1a1* promoter in mice resulted in increased production of acellular and cellular cementum and alveolar bone; however, this effect was lost when induction occurred in older tissues (Nottmeier et al., 2021). Over‐expression of Wnt1 was also associated with ectopic calcification in dental pulp as well as periodontal breakdown.

Sclerostin, encoded by the *Sost* gene, is a potent secreted Wnt inhibitor expressed by osteocytes and targets Wnt signaling in osteoblasts (Delgado‐Calle, Sato, & Bellido, 2017). Sclerostin loss‐of‐function is associated with sclerosteosis (OMIM# 269500) and dysregulation of sclerostin has been linked to Van Buchem Disease (OMIM# 239100), disorders of skeletal overgrowth. Sclerostin expression was detected in cementocytes of cellular cementum (Lehnen, Gotz, Baxmann, & Jager, 2012; van Bezooijen et al., 2009), and analysis of *Sost* ^*−/−*^ mice revealed thicker acellular cementum as well as more dramatic overgrowth of cellular cementum and alveolar bone, which suggests parallel roles for sclerostin in cementocytes and osteocytes (Figure 5r–w; Kuchler et al., 2014). Sclerostin also acts as a BMP antagonist and therefore represents an agent of potential cross‐talk between these signaling pathways.

Other gene‐editing approaches have reduced canonical Wnt signaling through varied approaches. Viral over‐expression of Wnt pathway inhibitor dickkopf‐1 (DKK1) decreased osteoblast markers and increased osteoclasts in the PDL. Changes in cementoblast signaling or cementum were not noted. Conditionally deleting the Wntless (*Wls*) gene (a G‐coupled receptor used in Wnt signaling) using the osteocalcin (*Ocn*) promoter resulted in reduced alveolar bone and cellular cementum, but progressive and widespread bone and tooth resorption make it difficult to specify changes in tissue production versus destruction (Lim et al., 2014; Yin et al., 2015).

## 7 CONCLUSIONS

The development of the periodontium is highly sensitive to agents regulating mineral metabolism on many levels. Disorders affecting the regulation of P_i_ or PP_i_ levels, loss‐of‐function of key ECM proteins, and derangement of major signaling pathways upstream of these factors, can all contribute to periodontal dysfunction. Alveolar bone responds in a similar fashion to mineralization disturbances as postcranial axial and appendicular bone, but it is more sensitive to some types of perturbations based on its rapid remodeling rate or other physiological differences. Cellular cementum generally reflects similar changes as alveolar bone, but acellular cementum is particularly sensitive to factors with direct roles in the biomineralization process, for example, TNAP, ANK, ENPP1, and BSP.

While many factors affecting periodontal mineralization have been identified, key questions remain about how mineralization is regulated in the cementum–PDL–bone periodontal complex. The tooth socket, or gomphosis, of mammals is a unique form of tooth attachment called thecodonty that is shared only with crocodilians among the extant vertebrates; in contrast, reptiles, amphibians, and fish feature a direct attachment of tooth to bone called acrodonty or pleurodonty (Gaengler, 2000). Substantial evidence has emerged in the last two decades from extant species and from the fossil record of nonmammalian synapsids (stem mammals) that collectively supports the early evolution of cementum as well as intermediate states of periodontal mineralization, that is, partially, variably, or wholly mineralized PDL that is effectively a state of ankylosis between the tooth and bone of attachment (Fong, LeBlanc, Berman, & Reisz, 2016; LeBlanc & Reisz, 2013; LeBlanc, Reisz, Brink, & Abdala, 2016; Luan et al., 2009; McIntosh et al., 2002). These findings in turn require evolution of regulatory factors and/or altered expression of factors that control mineralization in the periodontal complex to evolve the exquisitely regulated mammalian periodontium (Diekwisch, 2016).

In the periodontium, the tooth root is separated from the alveolar bone by the unmineralized PDL. This “sandwich” arrangement allows for many critical function of the PDL and periodontium: maintenance of flexibility of the tooth in the socket; distribution of occlusal forces; remodeling and repair of the PDL and alveolar bone initiated by directives from mechanoresponsive cells of the PDL; and neurovascular supplies to the periodontium. The PDL harbors osteo‐ and cemento‐progenitor and stem cells, is composed of a collagenous matrix nearly identical to those of bone and cementum, and is rich in pro‐mineralization factors (e.g., TNAP; Beertsen et al., 1997; B.L. Foster, Nociti Jr., & Somerman, 2013; Seo et al., 2004; Zweifler et al., 2015). The mechanism(s) by which ankylosis is avoided and the PDL remains unmineralized in the presence of neighboring mineralizing bone and cementum is an enduring mystery of periodontal mineralization. Some of the factors discussed in this review contribute in important ways to proper periodontal mineralization. However, even with the many genetic knockouts and double gene knockouts reported to date, the periodontal structure persists and the cementum–PDL–bone gomphosis remains patent, which points to critical roles of other factors not yet identified. This unanswered question of how the PDL persists between “a rock and a hard place” lies at the heart of the evolution of the periodontium, underpins all of periodontal biology, and is clinically relevant to understanding disease and regeneration of periodontal tissues. Ongoing research in the coming decades will provide new clues and produce exciting revelations about periodontal biology.

## CONFLICT OF INTEREST

The authors declare no potential conflicts of interest with respect to the research, authorship, and/or publication of this article.

## Data Availability

The data that support the findings of this study are available from the corresponding author upon reasonable request.
